# Knowledge distillation with ensembles of convolutional neural networks for medical image segmentation

**DOI:** 10.1117/1.JMI.9.5.052407

**Published:** 2022-05-28

**Authors:** Julia M. H. Noothout, Nikolas Lessmann, Matthijs C. van Eede, Louis D. van Harten, Ecem Sogancioglu, Friso G. Heslinga, Mitko Veta, Bram van Ginneken, Ivana Išgum

**Affiliations:** a Amsterdam University Medical Center, University of Amsterdam, Department of Biomedical Engineering and Physics, Amsterdam, The Netherlands; b Radboud University Medical Center, Department of Medical Imaging, Nijmegen, The Netherlands; c Eindhoven University of Technology, Department of Biomedical Engineering, Eindhoven, The Netherlands; d Amsterdam University Medical Center, University of Amsterdam, Department of Radiology and Nuclear Medicine, Amsterdam, The Netherlands; e Amsterdam University Medical Center, University of Amsterdam, Amsterdam Cardiovascular Sciences, Heart Failure & Arrhythmias, Amsterdam, The Netherlands; f University of Amsterdam, Informatics Institute, Amsterdam, The Netherlands

**Keywords:** knowledge distillation, ensembles, deep learning, segmentation

## Abstract

**Purpose:**

Ensembles of convolutional neural networks (CNNs) often outperform a single CNN in medical image segmentation tasks, but inference is computationally more expensive and makes ensembles unattractive for some applications. We compared the performance of differently constructed ensembles with the performance of CNNs derived from these ensembles using knowledge distillation, a technique for reducing the footprint of large models such as ensembles.

**Approach:**

We investigated two different types of ensembles, namely, diverse ensembles of networks with three different architectures and two different loss-functions, and uniform ensembles of networks with the same architecture but initialized with different random seeds. For each ensemble, additionally, a single student network was trained to mimic the class probabilities predicted by the teacher model, the ensemble. We evaluated the performance of each network, the ensembles, and the corresponding distilled networks across three different publicly available datasets. These included chest computed tomography scans with four annotated organs of interest, brain magnetic resonance imaging (MRI) with six annotated brain structures, and cardiac cine-MRI with three annotated heart structures.

**Results:**

Both uniform and diverse ensembles obtained better results than any of the individual networks in the ensemble. Furthermore, applying knowledge distillation resulted in a single network that was smaller and faster without compromising performance compared with the ensemble it learned from. The distilled networks significantly outperformed the same network trained with reference segmentation instead of knowledge distillation.

**Conclusion:**

Knowledge distillation can compress segmentation ensembles of uniform or diverse composition into a single CNN while maintaining the performance of the ensemble.

## 1 Introduction

Convolutional neural networks (CNNs) achieve state-of-the-art performance in many medical image analysis tasks,^1^ such as automatic detection, classification, and in particular segmentation.^2^ A large variety of different network architectures have been proposed of which some are now widely used, such as U-Net,^3^ residual neural network (ResNet),^4^ and CNNs with dilated convolutions.^5^ These architectures have different properties: the U-Net architecture uses upsampling and skip-connections to produce a high-resolution output, the ResNet architecture uses residual connections to construct very deep networks, and CNNs with dilated convolutions are distinguished by a particularly large receptive field with a limited number of parameters. It is well known that combining multiple networks into an ensemble often improves results compared to using a single CNN.^6^^–^^8^ Ensembles of CNNs have also won several recent segmentation challenges.^6^^,^^9^^,^^10^ However, even though ensembles often outperform a single network, they are more computationally expensive and time-consuming during inference. Due to their high computational demand and slow inference speed, ensembles are harder to deploy than single networks and can be less attractive for certain applications, such as clinical workstations.^11^

Techniques that reduce the footprint of CNN ensembles and other large and complex models are known as model compression techniques. A strategy for model compression is knowledge distillation, which refers to compressing the knowledge of a large and complex model and transferring it to a smaller, faster, and easier to deploy model without negatively impacting performance. Knowledge distillation is often explained with the analogy of a teacher teaching a student, where the large and complex model is referred to as the teacher model and the smaller model, to which the knowledge from the teacher model is transferred, is referred to as the student model.

Knowledge distillation from a large teacher model to a smaller student model was first proposed by Bucilă et al.,^12^ who trained compact neural networks with data automatically labeled by an ensemble of complex neural network classifiers trained for the classification of natural images. Ba and Caruana^13^ applied this approach to train shallower CNNs that mimic the function learned by deep CNNs and used it for natural image classification and speech recognition. Hinton et al.^14^ showed that an ensemble containing neural networks with identical architecture and trained procedure but with different parameter initialization can be compressed into a single neural network of a similar depth without performance loss. The single network was trained to predict both the class probabilities produced by the ensemble and the ground truth classification labels. The predicted class probabilities were used because they contain more information about the generalization performance of the ensemble compared to using predicted classes only.^14^ The method was applied to the classification of natural images and speech recognition. Chebotar and Waters^15^ showed that this technique can also be applied to ensembles of networks with different architectures for speech recognition.

Various other approaches for knowledge distillation that build on the work of Hinton et al.^14^ have been proposed and applied to different computer vision tasks such as natural image classification^16^^–^^18^ or segmentation.^19^ These studies focused on improving performance of the student network by adding extra loss terms to the distillation loss that focused on the similarity between intermediate network layers of the teacher model and the student model,^16^ the similarity between spatial attention maps of the teacher network and the student network,^17^ or the pair-wise pixel similarity between the output of the teacher and the student network.^19^ To surpass the need for the initial training of a strong teacher network,^18^ we proposed to train a teacher network as a single multi-branch network that served as an ensemble. The class distribution output of the teacher network was obtained by combining the output of all branches.

Knowledge distillation has also been used for a few applications in medical image analysis, such as classification,^20^^,^^21^ detection,^22^^,^^23^ and segmentation^8^^,^^24^^–^^29^ tasks. Knowledge distillation has been applied to obtain a smaller student network from a large teacher network such as a Google Inception V3 network for automatic detection of invasive cancer in whole slide images,^22^ a U-Net for automatic segmentation of neurons in microscope images^24^, or for liver^26^^,^^27^ and kidney^26^ segmentation in computed tomography images. An ensemble consisting of CNNs with the same architecture but trained with different training data has been combined with knowledge distillation to obtain a single CNN with a similar performance as the ensemble, which was then used for mitosis detection in histology images,^23^ and whole-brain segmentation in magnetic resonance imaging (MRI).^8^ Knowledge distillation has also been used to develop semi-supervised learning strategies^21^^,^^25^^,^^28^ where only a portion of the data is annotated and where the teacher network is used to obtained additional weak labels, aiming to distill more knowledge into a new network than just visible on the annotated training data. Knowledge distillation has also been combined with adversarial learning^29^ to increase the performance and robustness of the student network. In a federated learning context, knowledge distillation has been advocated as a strategy to preserve privacy but still allow for transfer of model knowledge between sites by training a model on the local data, but using knowledge distillation with the model outputs for public datasets to transfer the model knowledge.^30^

Furthermore, knowledge distillation has been used as a technique to enable multi-modal learning, such as from multiple MR sequences or entirely different modalities such as CT and MR. For instance, the knowledge of a model trained with multi-modal data has been distilled into models specialized on a specific modality or sequence.^31^^,^^32^ Knowledge distillation has also been used to enable learning from unpaired multi-modal data^33^ and to enable consistent multi-modal segmentation robust to missing modalities.^34^

### 1.1 Contributions

**Fig. 1:**
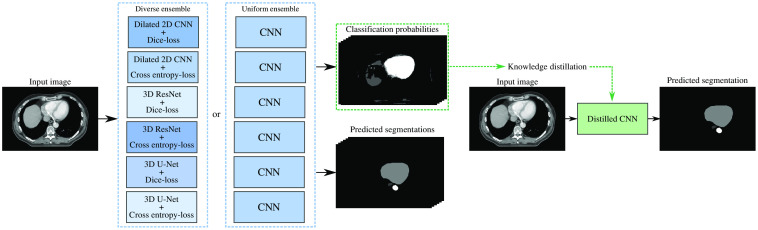
Schematics of the ensembling and knowledge distillation process for automatic segmentation of medical images. This paper uses an ensemble of six networks, which either have different architectures and different loss-functions (diverse ensemble), or the same architecture and the same loss-function (uniform ensemble). Subsequently, obtained classification probabilities from the ensemble are used to train the distilled network, which is a single, smaller network that performs the segmentation task more efficiently but has the same performance as the ensemble.

For medical image analysis tasks, knowledge distillation has the potential to enable the use of large ensembles to achieve better performance while maintaining the practical value of these algorithms and allowing for implementation in time-sensitive environments. However, there is a knowledge gap and this technique has only been studied for very specific applications, with teacher models consisting of only one large network,^22^^,^^24^ or an ensemble of networks with the same architecture but trained with different data.^8^^,^^23^ Ensembles can also consist of a diverse set of networks, such as networks differing in architecture, which might even further improve the performance of the ensemble. Therefore, this paper compares the application of knowledge distillation to two types of ensembles, namely, diverse ensembles of networks with different network architecture or trained with different loss-functions, and uniform ensembles of identical networks and training procedure but different random initialization. We apply knowledge distillation to each ensemble to obtain a single CNN, referred to as the distilled CNN, and test whether this network achieves a similar performance with substantially lower computational demand and faster inference compared to the ensemble (Fig. 1). Furthermore, we also compare the performance of the distilled network with the performance of a CNN with the same architecture but trained with reference segmentations alone, without the application of knowledge distillation. Instead of focusing on a single application, we perform these experiments for segmentation tasks in multiple datasets, differing in image modality, image dimensionality, and anatomical coverage.

## 2 Materials and Methods

### 2.1 Ensembles

Ensembles can consist of several networks with different architectures, or several networks with the same architecture. Either way, ensembles are most likely to outperform a single network when the individual networks in the ensemble make different mistakes as typically only a minority of the networks will make a specific mistake.^35^ Diversity can be established, for instance, by including different network architectures, using different loss functions during training of the separate networks, by initializing networks with different random seeds or by training networks with different data. We consider ensembles diverse if they differ in structure or training procedure, and we consider ensembles uniform if they differ only in initialization or training data.

A wide variety of CNN architectures and design principles are available for medical segmentation tasks. For the diverse ensemble that we evaluate in this paper, we selected three popular medical segmentation network architectures that differ in the number and type of network layers, connections between network layers, and kernels in convolutional layers. We use a three-dimensional (3D) CNN with an architecture inspired by U-Net, a 3D CNN with residual connections, and a two-dimensional (2D) CNN containing dilated convolutions. The diverse ensemble uses all three network architectures in two variants that are trained with different loss functions. One instance of each architecture is trained with a cross-entropy loss and the other with a soft Dice coefficient loss function. Hence, our diverse ensemble consists of six networks in total, differing in network architecture and loss-function used during training. The uniform ensemble consists of six instances of the same network architecture, each initialized with a different random seed to ensure a minimal level of diversity between the networks in the ensemble. Since the performance of network architectures could be task-dependent, the uniform ensemble uses the architecture that obtains the best performance for the task at hand. To obtain the output of an ensemble, the posterior classification probabilities predicted by the separate networks present in an ensemble are averaged.

#### 2.1.1 U-Net

**Fig. 2:**
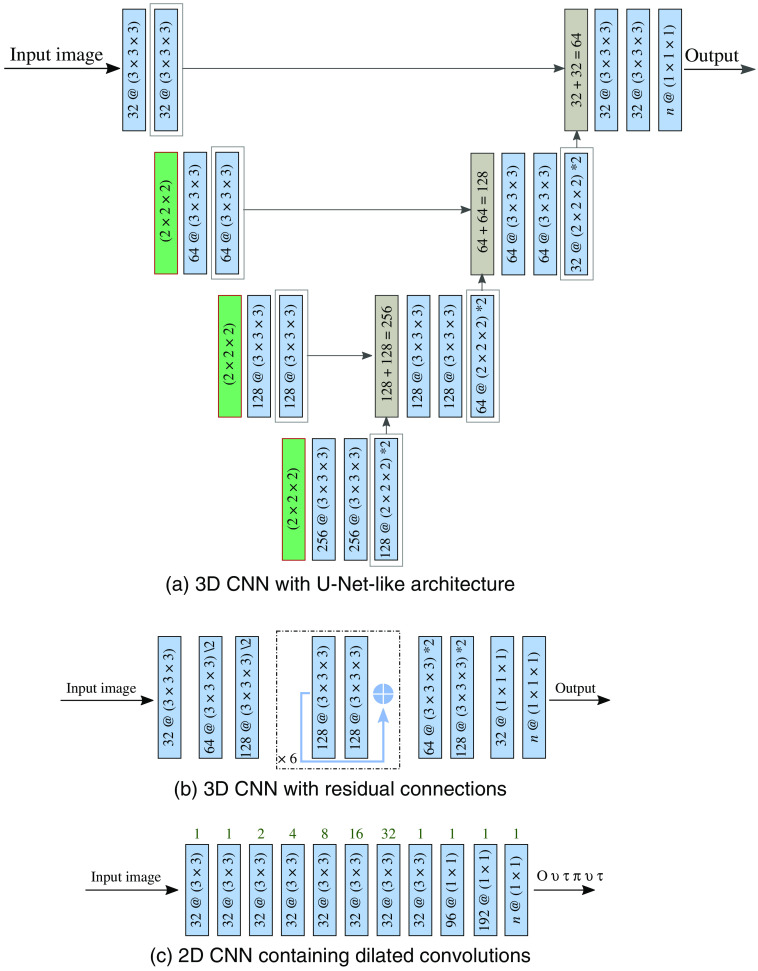
Architecture of the segmentation networks present in an ensemble. Blue blocks represent convolutional layers, green blocks represent max-pooling layers and gray blocks represent skip connection layers, consisting of an upsampling and a feature concatenation operation. For convolutional layers, the number of filters followed by (@) the size of the filters is given. For pooling layers, the size of the pooling area is given. For skip connections, the number of concatenated features is given. (a) A 3D CNN with a U-Net-like architecture. (b) A 3D CNN with residual connections, indicated by a light blue arrow. (c) A 2D CNN containing dilated convolutions with increasing dilation factors, indicated by green numbers above the shown network layers.

The U-Net^3^^,^^36^ network architecture in the ensemble is a 3D U-Net-like network with the typical compression and decompression path and skip connections [Fig. 2(a)]. Zero-padding is applied in all convolutional layers. All layers use the rectified linear units (ReLUs) for activation except for the output layer which uses the softmax activation function. Batch normalization is applied to stabilize training and reduce covariate shift.^37^

#### 2.1.2 ResNet

The ResNet^4^ network architecture in the ensemble is a 3D CNN with residual connections [Fig. 2(b)]. All convolutional layers apply zero-padding. ReLUs are used for activation in all layers, except the output layer which uses the softmax activation function. Batch normalization is applied in all layers except the dense and output layer to stabilize training and reduce covariate shift.^37^ Furthermore, dropout^38^ (p=0.5) is applied during training to reduce the risk of overfitting.

#### 2.1.3 Dilated network

The dilated network architecture in the ensemble is a 2D CNN with dilated convolution layers [Fig. 2(c)]. Dilated convolutions increase the receptive field of the network while keeping the number of parameters low. The proposed network architecture is inspired by the design of Yu and Koltun^5^ and the network used by Wolterink et al.^39^ ReLUs are used in all layers as activation function, except for the output layer which uses the softmax activation function. Batch normalization^37^ and dropout^38^ (p=0.5) are applied to the two dense layers to stabilize training and reduce covariate shift, and reduce the risk of overfitting.

### 2.2 Knowledge Distillation

The aim of knowledge distillation is to transfer the knowledge from the teacher model to a distilled model without performance loss. This distilled model is trained to mimic the output of the teacher model,^14^ which here is an ensemble of segmentation networks (Sec. 2.1). After training all networks in the ensemble, we pick the network architecture with best performance, i.e., the highest average Dice score on the validation set, for the architecture of the distilled model. The same architecture was also used in the uniform ensembles.

The distilled model is trained to mimic the output of the ensemble, specifically the average of the posterior classification probabilities that the individual networks in the ensemble predict for a sample. These averaged posterior classification probabilities, referred to as soft labels, are averaged per class and thus represent for each pixel in the image the probability distribution over all classes rather than just the class with highest probability. This probability distribution might contain information about the generalization performance of the ensemble and the ambiguity of different samples, and might thus allow the distilled student model to derive knowledge that is not contained in the manual reference segmentations, referred to as hard labels. These hard labels are additionally also supplied to the student model during training by combining a soft label loss $L_{s}$ with a hard label loss $L_{h}$ in the distillation loss

$$L_{d}=L_{s}+L_{h}.$$ (1)

We use the mean squared error between the softmax output of the distilled model and the soft labels for $L_{s}$, and the categorical cross-entropy between the output of the distilled model and the hard labels for $L_{h}$.

### 2.3 Evaluation

Evaluation of each separate network in an ensemble, the ensembles, and the distilled networks was performed by computing two different evaluation metrics. For each foreground class separately, the Dice coefficient was computed to evaluate the volume overlap between predicted and reference segmentations as

$$\text{Dice}(X_{C},Y_{C})=\frac{2|X_{C}\cap Y_{C}|}{\left|X_{C}|+|Y_{C}\right|},$$ (2)

where $X_{C}$ is the set of voxels in the image predicted by the model as part of class C and $Y_{C}$ is the set of foreground voxels of class C according to the reference standard.

Moreover, the average symmetrical surface distance (ASSD) was computed for evaluation of the borders of predicted segmentations. This metric expresses the average minimal distance of each point on the surface of the segmentation result to the surface of the reference segmentation, and vice versa, and is computed as

$$\mathrm{ASSD}(X_{C},Y_{C})=\frac{1}{2}(\mathrm{ASD}(X_{C},Y_{C})+\mathrm{ASD}(Y_{C},X_{C})),$$ (3)

with

$$\mathrm{ASD}(X,Y)=\frac{\underset{x\in X}{\sum }\;\min_{y\in Y}\|x-y\|}{\left|X\right|},$$ (4)

where $X_{C}$ and $Y_{C}$ and sets of points on the surface of the predicted and the reference segmentations for class C, respectively.

Throughout the manuscript, we sometimes report also the Hausdorff distance (HD), which was used in some of the segmentation challenges that we included in our evaluation. The HD expresses the maximum of all distances between points on surface X to the closest point on surface Y, and vice versa, and was thus calculated as

$$\mathrm{HD}(X_{C},Y_{C})=\max(h(X_{C},Y_{C}),h(Y_{C},X_{C})),$$ (5)

with

$$h(X,Y)=\underset{x\in X}{\max}\;\underset{y\in Y}{\min}\|x-y\|.$$ (6)

The Wilcoxon signed-rank test was used to test for significant differences in performance of each separate network in an ensemble and the ensemble, a distilled network and an ensemble, and a distilled network and the best performing network present in an ensemble, using a cut off of 0.005, which corresponds to 0.05 corrected for multiple testing on each dataset.

## 3 Data

In this study, three public datasets have used the segmentation of thoracic organs at risk (SegTHOR) dataset, the brain dataset, and the automated cardiac diagnosis challenge (ACDC) dataset. The SegTHOR dataset consists of radiotherapy treatment CT scans of the chest, while the brain dataset consists of brain MRI, and the ACDC dataset consists of cardiac cine-MRI. We deliberately selected datasets that differ in image modality (CT with and without contrast enhancement, MRI, and cine-MRI) and delineated anatomy (chest, brain, and heart).

### 3.1 Radiotherapy Chest CT (SegTHOR)

**Fig. 3:**
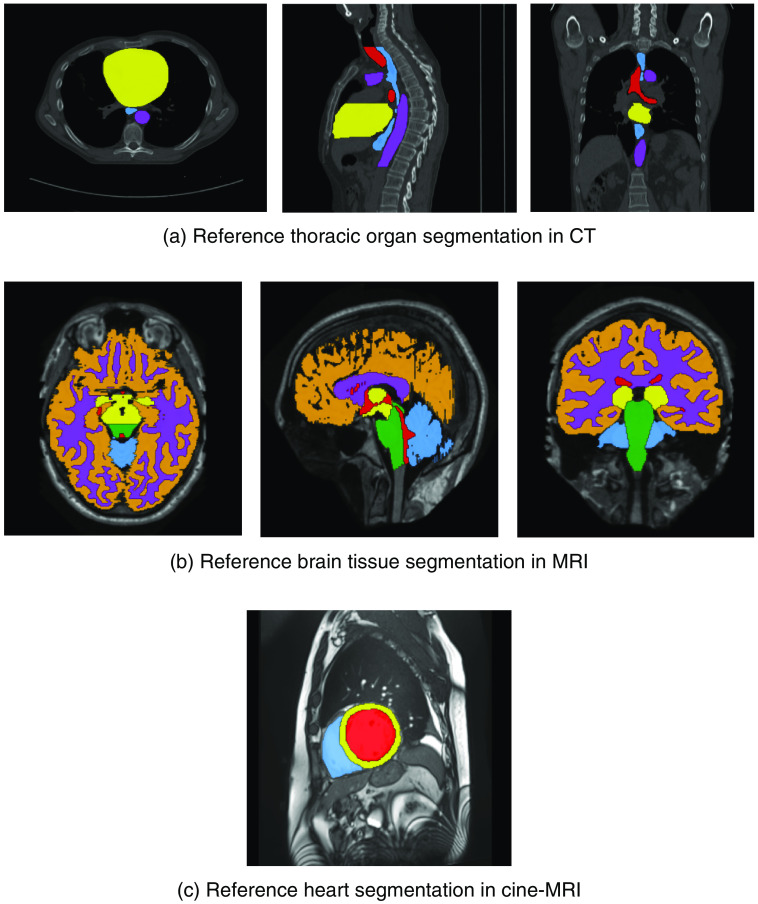
Example of the three datasets that were used to train and evaluate ensembles and knowledge distillation. (a) An axial, sagittal, and coronal slice from a CT scan, provided by the ISBI 2019 challenge on SegTHOR in CT images, in which the corresponding reference segmentation of the aorta (purple), trachea (red), heart (yellow), and esophagus (blue) are shown. (b) An axial, sagittal, and coronal slice from an MRI, provided by the MICCAI 2012 challenge on multi-atlas labeling in brain MRI, in which the corresponding reference segmentation of the cGM (orange), BS (green), WM (purple), vCSF (red), BGT (yellow), and CB (blue) are shown. (c) Short-axis view of the heart from an end-diastole cine-MRI, provided by the MICCAI 2017 automated cardiac diagnosis challenge, in which the corresponding reference segmentation of the left ventricle (red), myocardium (yellow), and right ventricle (blue) are shown.

The dataset contains 60 thoracic CT scans of patients that were referred for curative-intent radiotherapy for non-small cell lung cancer and was provided by the International Symposium on Biomedical Imaging (ISBI) 2019 challenge on SegTHOR in CT images.^40^ Scans were acquired with or without intravenous contrast injection, had an in-plane voxel size ranging from 0.90 to 1.37 mm, and a slice thickness ranging from 2 to 3.7 mm. For each scan in the dataset, manual reference segmentations of the aorta, trachea, heart, and esophagus were available [Fig. 3(a)]. The dataset was divided by the challenge organizers into a training dataset containing 40 scans, and a test dataset containing 20 scans.

### 3.2 Brain MRI

The dataset contains 35 brain MRI of patients aged 23±4.1 years and was introduced in the Medical Image Computing and Computer Assisted Intervention (MICCAI) 2012 challenge on multi-atlas labeling in brain MRI.^41^ MR images originate from the OASIS project.^42^ Images were acquired in the sagittal plane with a Siemens Vision 1.5 T scanner. Slices had an isotropic in-plane voxel size of $1\;{\mathrm{mm}}^{2}$ and a slice thickness of 1.25 mm. Subsequently, images were resized to an isotropic voxel size of $1\;{\mathrm{mm}}^{3}$. Marcus et al.^42^ and the OASIS website^43^ provide a detailed description of the image acquisition. For each MRI, reference segmentations were available for 134 tissue classes, which were merged into six different tissue classes by Moeskops et al.:^44^ the cortical gray matter (cGM), brain stem (BS), white matter (WM), ventricular cerebrospinal fluid (vCSF), basal ganglia and thalami (BGT), and cerebellum (CB) [Fig. 3(b)]. The dataset was divided by the MICCAI challenge organizers into a training dataset containing 15 scans and a test dataset containing 20 scans.

### 3.3 Cardiac Cine-MRI (ACDC)

The dataset contains the cine-MRI of 150 patients acquired for clinical analysis. The set was introduced by the MICCAI 2017 ACDC.^45^ Patients were divided into one of five disease categories: healthy, systolic heart failure with infarction, dilated cardiomyopathy, hypertrophic cardiomyopathy, or abnormal right ventricle. MR images were obtained with a 1.5 T Siemens or a 3.0 T Siemens scanner. Images were acquired during breath hold with a conventional SSFP sequence. Whole short-axis slices covering the left ventricle were acquired. Slices had an isotropic in-plane resolution ranging from 1.34 to 1.68 mm, and a slice thickness ranging from 5 to 10 mm with sometimes an inter-slice gap of 5 mm. For each patient end-systole and end-diastole frames were available. However, in this study, only the end-diastole frames were used. The dataset was divided by the challenge organizers into a training dataset containing 100 scans, and a test dataset containing 50 scans. For each scan in the training dataset, manual reference segmentations of the left ventricle, myocardium, and right ventricle were provided [Fig. 3(c)].

## 4 Experiments and Results

Before performing any experiments, all training datasets as provided by the challenges were randomly divided into a training set and validation set used for method development. These subsets contained 35 training and five validation scans for the SegTHOR dataset, 13 training and 2 validation scans for the Brain dataset, and 90 training and 10 validation scans for the ACDC dataset. Only for final evaluation, the hold-out test sets as provided by the organizers of the challenges were used. The networks were implemented in Python using PyTorch^46^ on a NVIDIA 2080 Ti with 11 GB of memory.

### 4.1 Training Procedure

For each of the three datasets, 11 separate networks were trained. These were six networks for the diverse ensemble, consisting of two 3D CNNs with a U-Net-like architecture, two 3D CNNs with residual connections, and two 2D dilated CNNs, each trained once with the Dice coefficient loss function and once with the cross-entropy loss. For the uniform ensemble, the best performing network was trained five more times with each model initialized with a different random seed.

For each network, network parameters were optimized for up to 100,000 iterations, using Adam^47^ with a fixed learning rate of 0.001. The networks were evaluated every 10,000 iterations on the full validation set until the validation loss did not decrease anymore. All networks were trained with mini-batches containing randomly chosen sub-images (patches) with all tissue classes equally represented. Additionally, data augmentation was employed by rotating each sub-image in a mini-batch with a random rotation angle between −10  deg and +10  deg.

During training of CNNs with the U-Net-like architecture, at every iteration, a mini-batch containing 4 randomly sampled 3D 72×72×72 sub-images was shown to the networks. During training of CNNs with residual blocks, at every iteration, a mini-batch containing 12 randomly sampled 3D 64×64×64 sub-images was shown to the networks. During inference, full images were analyzed.

During training of 2D dilated networks, at every iteration a mini-batch containing 40 randomly sampled 2D 186×186 sub-images extracted from the axial, coronal, or sagittal plane, was shown to the networks, which classified the 55×55 voxels in the center of the sub-images. Networks were trained to classify each voxel in a scan based on the analysis of three orthogonal slices. During inference, all slices from the axial, coronal, and sagittal planes of a scan were analyzed. This resulted in three 3D multi-class probability maps, which were subsequently averaged to obtain a final probability map. Finally, each voxel was assigned the class with the highest class probability.

### 4.2 Individual Networks

#### 4.2.1 Radiotherapy chest CT

**Fig. 4:**
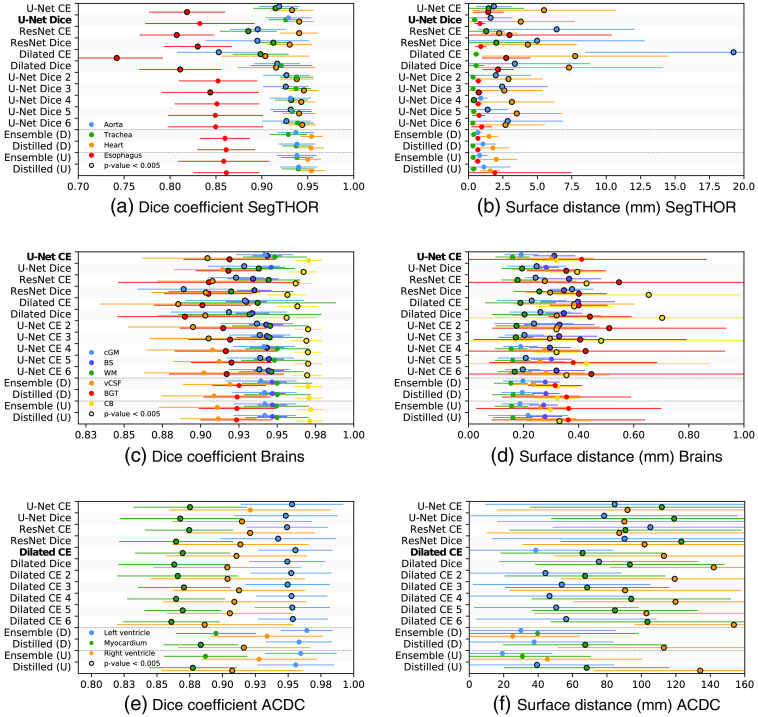
Results for the hold-out test sets of the three datasets (a,b: SegTHOR; c,d: Brains; e,f: ACDC) obtained with individual networks and uniform (U) and diverse (D) ensembles and the corresponding distilled networks. The network architecture used in the uniform ensemble is indicated in bold. In each line, a dot indicates the average across the test set while the corresponding horizontal line indicates the standard deviation. Significance outcome by the Wilcoxon signed-rank test compared to the performance of the diverse ensemble with the top six networks and the corresponding distilled network, and the uniform ensemble with the networks with the same architecture and the corresponding distilled network is indicated by a black circle (i.e., p<0.005).

Initially, to deal with varying voxel sizes, images were resampled to an isotropic in-plane voxel size of 0.98 mm and a slice thickness of 2.5 mm, which was the most common voxel size present in the dataset. During inference, results were resampled to the original image resolution. Preliminary experiments showed that using the cross-entropy as a loss function led to more segmentation errors. Therefore, during training of separate networks with the cross-entropy loss, errors made on a foreground class were penalized 10 times stronger than errors made on the background class. Furthermore, all sub-images in a mini-batch contained at least one foreground voxel. Results obtained on the test dataset are shown in Figs. 4(a) and 4(b). Overall, the best performance was achieved with the U-Net-like network trained with Dice coefficient loss function. Five additional instances of this network were initialized with different random seeds and trained with otherwise identical training settings to form a uniform ensemble. Differences in obtained Dice coefficients and ASSDs between the six U-Net-like networks trained with the Dice coefficient as loss-function were below 0.02 and 1.20 mm, respectively [Figs. 4(a) and 4(b)].

#### 4.2.2 Brain MRI

Results of automatic segmentation of the six brain tissue classes in the independent test set are shown in Figs. 4(c) and 4(d). Overall, the U-Net-like network trained with the cross-entropy loss obtained the best performance. Five additional instances of this network were initialized with different random seeds and trained with otherwise identical training settings. Differences in obtained Dice coefficients and ASSDs between the six U-Net-like networks trained with the cross-entropy loss were below 0.01 and 0.17 mm, respectively [Figs 4(c) and 4(d)].

#### 4.2.3 Cardiac cine-MRI

Contrary to the brain and SegTHOR datasets, the ACDC dataset contains images with a very large slice thickness compared to the in-plane voxel size. Therefore, only 2D axial slices of MR images were analyzed. The architectures of the two ResNets the two U-Net-like networks were adapted to analyze only 2D images instead of 3D images by replacing 3D convolutions with 2D convolutions. Results are shown in Figs. 4(e) and 4(f). Because overall the best performance was obtained with the dilated network trained with cross-entropy loss, five additional instances were initialized with different random seeds and trained with otherwise identical training settings. Differences in obtained Dice coefficients and HDs between the six dilated networks trained with the cross-entropy loss were below 0.03 and 41 mm, respectively [Figs. 4(e) and 4(f)].

### 4.3 Uniform and Diverse Ensembles

**Fig. 5:**
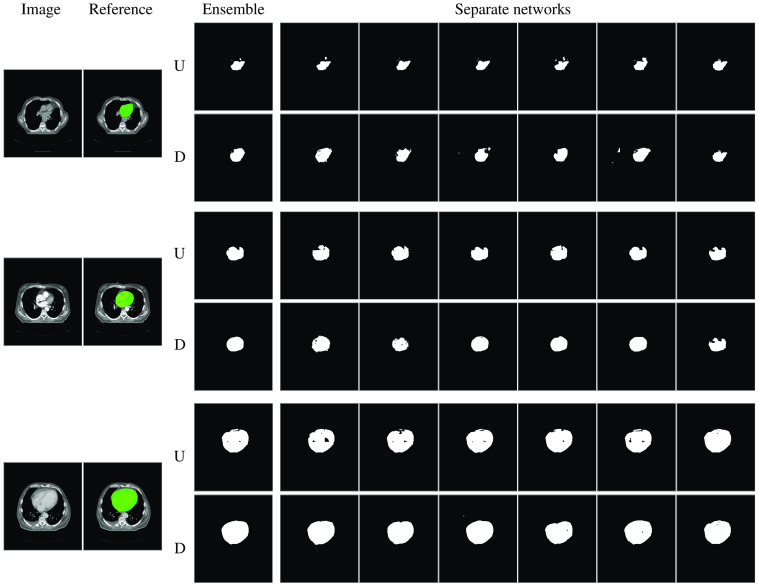
Automatic heart segmentation results for three different scans from the SegTHOR test dataset. The six right-most columns show the automatic segmentations obtained with the individual networks in the uniform (U) and diverse (D) ensemble. The uniform ensemble contained six U-Nets, trained with the Dice coefficient as loss-function but with different random seeds, while the diverse ensemble contained two dilated networks, two ResNets, and two U-Nets, trained with the cross-entropy or the Dice coefficient as loss-function. The right-most column shows the results obtained with the network that is part of both the uniform and the diverse ensemble, i.e., the segmentations are identical.

Segmentation results obtained by the uniform and diverse ensembles for various anatomical structures in the three test datasets are shown in Fig. 4. Both the diverse and the uniform ensemble performed better than any of the individual networks. However, there was no substantial difference in the performance of the uniform and diverse ensembles themselves. Figure 5 shows automatic segmentations in three different scans of the test dataset, obtained with the diverse and uniform ensemble and the separate networks present in the ensembles. Networks present in the diverse ensemble made errors in different areas compared to networks present in the uniform ensemble.

### 4.4 Ensembles Distilled into a Single Network

The performance of separate networks varied between the datasets, i.e., there was not a single network that performed best across all datasets. For each ensemble, the network architecture that obtained the highest Dice coefficients and lowest distance errors, averaged over all classes, was used as the preferred network architecture for the distilled network (indicated in bold letters in Fig. 4). This enables a direct comparison between the distilled network and the best performing network present in the ensemble. For each dataset, two distilled networks were trained: one from the diverse ensemble and one from the uniform ensemble. These networks were trained with the combined loss function that includes soft and hard labels [Eq. (1)] and the network parameters were initialized with those of the best performing network in the ensemble.

Across all three datasets, the distilled networks achieved better segmentation performance than the individual networks that were trained only with reference segmentations (Fig. 4). Except for some tasks in the ACDC dataset, the distilled networks also reached the performance of the ensembles they were derived from. Similar to the minor differences between uniform and diverse ensembles, networks derived from these different types of ensembles also performed very similar to each other, with networks derived from diverse ensembles performing minimally better overall.

For each dataset, Table 1 lists the difference at inference between an ensemble and its corresponding distilled network with respect to network size, GPU memory usage, and average analysis time per scan. Distilled networks contained 4 to 89 times fewer trainable parameters, needed 4 to 10 times less GPU memory at inference, and were 5 to 8 times faster compared to the ensembles.

**Table 1:** Difference in network size in terms of the number of trainable parameters, GPU memory usage, and the average time needed to process one scan. Compared are a diverse ensemble (D), a uniform ensemble (U), and their corresponding distilled networks for three different datasets (SegTHOR, brain, and ACDC).

	Trainable parameters	GPU memory requirement (GB)	Inference time per scan (s)
SegTHOR ensemble (D)	$23.17\times 10^{6}$	14.00	602.2±143.0
SegTHOR distilled (D)	$5.64\times 10^{6}$	1.37	123.0±28.8
SegTHOR ensemble (U)	$33.84\times 10^{6}$	8.22	738.0±172.8
SegTHOR distilled (U)	$5.64\times 10^{6}$	1.37	123.0±28.8
Brain ensemble (D)	$23.20\times 10^{6}$	6.10	312.8±21.0
Brain distilled (D)	$5.64\times 10^{6}$	1.10	59.9±4.9
Brain ensemble (U)	$33.84\times 10^{6}$	6.60	359.4±29.4
Brain distilled (U)	$5.64\times 10^{6}$	1.10	59.9±4.9
ACDC ensemble (D)	$7.97\times 10^{6}$	5.44	16.7±1.9
ACDC distilled (D)	$0.09\times 10^{6}$	1.36	2.1±0.3
ACDC ensemble (U)	$0.54\times 10^{6}$	8.16	12.6±1.8
ACDC distilled (U)	$0.09\times 10^{6}$	1.36	2.1±0.3

### 4.5 Ablation Study

**Fig. 6:**
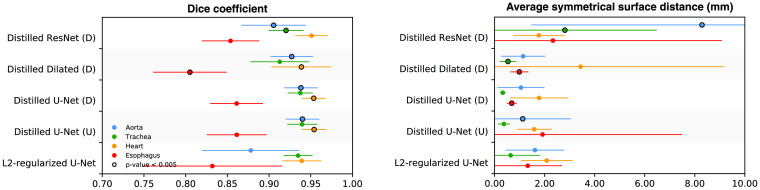
Segmentation performance on the SegTHOR test set obtained with different distilled networks, derived from diverse (D) and uniform (U) ensembles. Additionally, results obtained with a U-Net-like architecture trained with the Dice coefficient as loss-function and additional L2-regularization are shown. In each line, a dot indicates the average over the test set while the corresponding horizontal line shows the standard deviation. The performance of the distilled ResNet and the distilled dilated network is compared to the performance of the distilled U-Net-like network from the diverse ensemble, while the performance of the two distilled U-Net-like networks is compared to the performance of the L2-regularized network. Significance in these comparisons by the Wilcoxon signed-rank test is indicated with a black circle (i.e., p<0.005).

Using the SegTHOR dataset, we investigated whether it is beneficial to use the architecture of the best performing network of an ensemble as architecture of the distilled network. We trained two additional distilled networks using the two other network architectures from the ensemble, namely the architecture containing residual connections and the architecture containing dilated convolutions, and compared their performance with the distilled network using the architecture of the best performing network, namely, the U-Net-like architecture. Results are shown in Fig. 6. Overall, when using the best performing individual architecture for the distilled network, better performance was achieved compared to using one of the other two architectures for the distilled network.

Additionally, knowledge distillation can be seen as a form of regularization, where the distillation loss used during training of a distilled network discourages the prediction of probabilities if they do not agree with the prediction of the ensemble. Similarly, L2-regularization discourages the network to obtain large network weights during training and it is commonly used as regularization technique for training CNNs. To investigate whether knowledge distillation is more beneficial than simple L2-regularization, we trained a network with U-Net-like architecture and Dice coefficient loss, which were the settings used for the distilled network, and L2-regularization instead of the soft label loss term. Results are shown in Fig. 6. For all organs of interest, automatic segmentation with a distilled network obtained better Dice coefficients and ASSDs compared to automatic segmentation with a U-Net trained with L2-regularization.

### 4.6 Comparison with Other Methods

Previously, several methods have been proposed for automatic segmentation of organs at risk in the SegTHOR dataset, brain tissue in the brain dataset, and cardiac structures in the ACDC dataset. The purpose of this paper was not to present a new state-of-the-art segmentation method for any of these datasets, but to investigate how well the concept of knowledge distillation works across different datasets and with different types of ensembles. However, we still compared the performance of the ensembles and the networks derived with knowledge distillation with a number of previously presented methods to demonstrate that the ensembles that our ensembles achieved comparable performance.

#### 4.6.1 Radiotherapy chest CT

We compare the performance of our two ensembles and the corresponding distilled networks with the five best performing methods that participated in the SegTHOR challenge. Four of these methods also employed an ensemble of CNNs. Van Harten et al.^7^ employed an ensemble consisting of a 2D dilated network and a 3D ResNet. Wang et al.^48^ created an ensemble consisting of three cascaded V-Nets and averaged the output of networks preserved during the last five training iterations, while Han et al.^49^ trained multiple instances of two single-class cascaded V-nets. He et al.^50^ used an ensemble of multi-task U-Net like networks performing simultaneous classification and segmentation. Finally, Zhang et al.^51^ used two cascaded V-Nets which shared skip connections between corresponding convolution blocks.

For postprocessing, previous methods employed largest component selection, with or without data-specific rules, to improve their results.^7^^,^^48^^–^^51^ Therefore, for comparison, we also postprocessed our results obtained by our two ensembles and the corresponding distilled networks in the same way. Table 2(a) lists the results as reported in previous work and our postprocessed results. The results show that our ensembles and distilled networks obtain Dice coefficients and HDs which were close to the results of the best performing method. Overall, our diverse ensemble and distilled network obtain slightly better results compared to their uniform counter parts. For segmentation of the aorta, heart, trachea, and esophagus differences in Dice coefficients between the distilled network from the diverse ensemble and the best performing method were <0.01, while differences in HDs were below 0.10 mm, which is less than a voxel in size.

**Table 2:** Results obtained on (a) SegTHOR test dataset, (b) brain test dataset, and (c) ACDC test dataset.

(a) SegTHOR dataset												
	Aorta		Trachea		Heart		Esophagus					
	Dice	HD	Dice	HD	Dice	HD	Dice	HD				
van Harten et al.^7^	0.93	2.7	0.91	2.1	0.94	2.0	0.84	3.4				
Wang et al.^48^	0.94	0.16	0.92	0.21	**0.95**	0.16	0.86	0.29				
Han et al.^49^	**0.95**	0.12	**0.93**	**0.15**	**0.95**	**0.13**	**0.87**	**0.26**				
He et al.^50^	**0.95**	**0.11**	0.92	0.18	**0.95**	0.14	0.86	0.27				
Zhang et al.^51^	0.92	0.35	0.89	0.57	0.93	0.63	0.84	0.40				
Ensemble (D)	0.93	0.25	0.91	0.22	0.94	0.20	0.86	0.27				
Distilled (D)	0.94	0.22	0.92	0.23	0.94	0.18	0.86	0.30				
Ensemble (U)	0.93	0.24	0.92	0.21	0.94	0.20	0.84	1.07				
Distilled (U)	0.94	0.24	0.92	0.23	0.94	0.18	0.83	1.98				
(b) Brain dataset												
	cGM		BS		WM		vCSF		BGT		CB	
	Dice	MSD	Dice	MSD	Dice	MSD	Dice	MSD	Dice	MSD	Dice	MSD
Moeskops et al.^44^	0.91	0.39	0.93	0.46	0.93	0.28	0.86	0.52	0.86	0.72	0.95	0.61
Moeskops et al.^52^	**0.94**	—	0.93	—	**0.95**	—	0.88	—	0.91	—	0.96	—
Ensemble (D)	**0.94**	**0.19**	**0.95**	**0.25**	**0.95**	**0.16**	**0.92**	**0.17**	**0.93**	**0.30**	**0.97**	**0.24**
Distilled (D)	**0.94**	0.20	**0.95**	0.26	**0.95**	0.18	0.91	0.28	0.92	0.38	**0.97**	0.26
Ensemble (U)	**0.94**	**0.19**	**0.95**	0.26	**0.95**	0.17	0.91	0.24	0.92	0.40	**0.97**	0.25
Distilled (U)	**0.94**	0.25	**0.95**	0.27	**0.95**	0.19	0.91	0.30	0.92	0.40	**0.97**	0.31
(c) ACDC dataset												
	Left ventricle		Myocardium		Right ventricle							
Method	Dice	HD	Dice	HD	Dice	HD						
Isensee et al.^53^	0.95	7.15	**0.91**	8.70	0.92	11.13						
Calisto and Lai-Yuen^54^	0.96	**5.59**	0.87	**8.20**	**0.94**	**10.18**						
Baumgartner et al.^55^	0.96	6.53	0.89	8.70	0.93	12.67						
Khened et al.^56^	0.96	8.13	0.89	9.84	**0.94**	13.99						
Zotti et al.^57^	0.96	6.18	0.89	9.59	0.93	11.05						
Painchaud et al.^58^	0.96	6.15	0.88	8.65	0.93	13.72						
Ensemble (D)	**0.97**	6.68	0.90	8.94	**0.94**	11.53						
Distilled (D)	0.96	7.84	0.89	11.10	0.93	14.72						
Ensemble (U)	0.96	7.64	0.89	10.29	0.93	11.73						
Distilled (U)	0.96	8.97	0.88	10.71	0.92	15.41						

Results for the aorta, trachea, heart, and esophagus in the SegTHOR dataset, the cortical gray matter (cGM), brain stem (BS), white matter (WM), ventricular cerebrospinal fluid (vCSF), basal ganglia and thalami (BGT), and cerebellum (CB) in the brain dataset, and the left ventricle, myocardium, and right ventricle in the ACDC dataset are shown separately for different evaluation metrics: the Dice coefficient (Dice), and the HD in mm or MSD in mm.

#### 4.6.2 Brain MRI

We compare the performance of our two ensembles and the corresponding distilled networks with two earlier methods that performed segmentation of the six merged tissue classes in the brain MRI dataset. None of these methods employed an ensemble of CNNs. For automatic segmentation of brain tissue in the brain dataset, Moeskops et al.^44^ used a multi-scale CNN. Later, Moeskops et al.^52^ used a 2D dilated network that was trained with an adversarial loss.

Table 2(b) lists the results obtained with our two ensembles and the corresponding distilled networks on the Brain dataset and compares them with results as reported in previous work. For all tissue classes, our ensembles and distilled networks obtain better or the same Dice coefficients compared to earlier methods [Table 2(b)]. Simultaneously, for every tissue class, our ensembles and distilled networks obtain lower mean surface distances (MSD). Overall, for all tissue classes, our diverse ensemble obtained the highest Dice coefficients and lowest MSD.

#### 4.6.3 Cardiac cine-MRI

We compare the performance of our two ensembles and the corresponding distilled networks with the six best performing methods that participated in the ACDC challenge. Two of these methods also employed an ensemble of CNNs. For automatic segmentation of cardiac structures in the ACDC dataset, Isensee et al.^53^ used an ensemble consisting of a 2D and 3D U-Net while Calisto and Lai-Yuen^54^ used an ensemble consisting of a 2D and 3D network with hyperparameters optimized for the specific segmentation task. Baumgartner et al.^55^ used a single 2D U-Net. Khened et al.^56^ first extracted a region of interest with Fourier analyses after which a dense-Net analyzed the region. Zotti et al.^57^ used a multi-resolution U-Net and additionally incorporated a cardiac shape prior. Painchaud et al.^58^ used a U-Net to obtain segmentation predictions of the cardiac structures, which were subsequently converted into anatomically correct ones using an adversarial variational auto-encoder.

Similar to methods proposed for automatic segmentation of organs at risk in the SegTHOR dataset (Sec. 4.6.1), most previously proposed methods perform selection of the largest component, with or without data-specific rules, to improve results.^54^^–^^56^^,^^58^ Therefore, for comparison, we also postprocessed our results obtained by the two ensembles and the corresponding distilled networks in the same way. They are given in Table 2(c).

For automatic segmentation of the left ventricle, our diverse ensemble obtained the highest Dice coefficient [Table 2(c)]. For all other structures, Dice coefficients obtained with our two ensembles and the corresponding distilled networks were similar or close to the best performing method in the challenge, with our diverse ensemble and distilled network slightly outperforming the uniform ensemble and distilled network, respectively. For segmentation of the left ventricle, the myocardium, and the right ventricle differences in Dice coefficients between the distilled network and the best performing method were <0.03, while differences in HDs were <5.23  mm.

## 5 Discussion

Knowledge distillation aims to reduce the footprint of large models such as ensembles which are often used for segmentation tasks in medical imaging. This work shows that ensembles consisting of networks with different architectures and loss-functions or networks with the same architecture and loss-function achieve excellent performance across several segmentation tasks. Additionally, this work shows that knowledge distillation can be used to compress a diverse or uniform ensemble into a single, distilled network which deploys more easily compared to the ensemble due to a lower computational demand and a higher computational speed.

Due to the high number of images that need to be processed on a daily basis, a fast inference with a low computational demand can be very important requirements for method deployment in the clinic.^11^ Therefore, this work focused on deployability of ensembles for segmentation of structures in medical images. Although training multiple networks for an ensemble demands more time compared to training a single network, training is performed only during development whereas inference will be performed every time the final network is deployed. Hence, this paper aimed to investigate ways to perform image segmentation while decreasing computational demand and increasing inference speed of successful approaches such as ensembles.

This paper investigated the application of knowledge distillation on two types of ensembles: a diverse ensemble containing networks that differed in architecture and loss-function, and a uniform ensemble containing network with the same architecture and loss-function but initialized with different random seeds. To ensure diversity among network architectures, three popular medical image segmentation network architectures were chosen that differed in the number and type of network layers, connections between network layers, and kernels in convolutional layers. These network architectures were a U-Net inspired architecture, a 3D CNN with residual connections, and a 2D CNN containing dilated convolutions, which were included in the diverse ensemble. In recent years, all three network architectures have shown a good performance for medical image segmentation tasks.^7^^,^^39^^,^^50^^,^^52^^,^^53^^,^^55^^,^^57^^,^^58^ Other network architectures, such as DeepLabV3 with atrous spatial pyramid pooling^59^ or multi-scale CNNs^44^^,^^60^ could also have been included in our ensemble to increase diversity or to obtain a larger ensemble. Increasing the number of networks in an ensemble might also lead to a better performance and would therefore be an interesting topic for future research.

The uniform and diverse ensemble, and subsequent knowledge distillation were evaluated using three different datasets: the SegTHOR dataset, in which four organs were segmented in contrast enhanced and non-contrast enhanced chest CT scans,^40^ the brain dataset, in which six different brain structures were segmented in brain MRI,^41^^,^^42^ and the ACDC dataset, in which three different heart structures were segmented in cardiac cine-MRI.^45^ Results show that for all three datasets, the uniform and diverse ensembles outperformed individual networks present in those ensembles. For the SegTHOR and ACDC dataset, the diverse ensembles obtained slightly better performance compared to the uniform ensembles but results were not always significant. Results also showed that for all three datasets, different networks performed best for the different tasks. There was no specific architecture trained with a specific loss-function that performed best on all three datasets. Therefore, for each dataset the best performing architecture was chosen based on obtained results and used as the preferred architecture for the networks in the uniform ensemble. Furthermore, this network architecture was also used as architecture for the distilled networks, which enabled a direct comparison between the best performing network in the ensemble and the distilled network. Using the SegTHOR dataset, it was additionally shown that employing other network architectures for the distilled network resulted in a significantly lower performance of the distilled network compared to using the best performing architecture from an ensemble.

The output of ensembles was obtained by averaging the posterior classification probabilities predicted by the separate networks. Besides averaging the predictions of the separate networks, different forms of combining the predictions could be considered, such as weighted averaging or majority voting. In this study, networks in an ensemble were trained using identical datasets during training. Ensembles are most likely to outperform a single network when the networks present in the ensemble are diverse and can compensate for errors made by some of them. Therefore, using different subsets of the training dataset to train separate networks might also lead to diverse networks. Hence, in future work, we will investigate whether combining predictions of separate networks in a different way or training separate networks on different subsets of the training data leads to a better performance of the ensemble.

For the SegTHOR and brain dataset, knowledge distillation led to a single, smaller network that performed the same or better compared to the corresponding ensemble. For the ACDC dataset, compared to the corresponding ensemble, the distilled network from the diverse ensemble performed the same for segmentation of the left ventricle, while the distilled network from the uniform ensemble performed the same for segmentation of the right ventricle. For other structures, the ensembles outperformed the distilled networks. Inspection of automatic segmentations of images in the ACDC dataset obtained with the ensembles and distilled networks showed that most differences did not occur between foreground classes but near the border of foreground classes and the background. Showing more examples of the border between foreground classes and the background during training of the distilled network could improve the performance of the distilled network. Nonetheless, for all three datasets, both distilled networks obtained significantly better results compared to the best performing network present in the corresponding ensemble, showing the added value of the distillation step.

During knowledge distillation, soft labels were considered as the knowledge to be transferred from the ensemble to the distilled network. Soft labels contain information about the generalization performance of an ensemble. It could be argued that besides soft labels, features from intermediate layers of separate networks could also be considered as transferable knowledge and thus should be mimicked by the distilled network. However, for this, it needs to be ensured that the features of interest are the same size between the separate networks in the ensemble and the distilled network, which could make training more complex when the ensemble consists of networks with different architectures.

The knowledge distillation loss discourages predicted probabilities from the distilled network if they do not agree with the prediction of the ensemble, i.e., the prediction of the ensemble is used as a boundary to guide the distilled network during training and might therefore act as a form of regularization. Similarly, L2-regularization essentially discourages a network to obtain large network weights, i.e., it enforces a boundary on the network weights. Using the SegTHOR dataset, we therefore, compared knowledge distillation with L2-regularization which is a popular regularization method for training CNNs. Results showed that the distilled networks outperformed the same network trained with additional L2-regularization. Other regularization techniques, such as Dropout and data augmentation, could also have been investigated. However, Dropout and augmentation by random image rotation were already applied during training of the separate networks. Hence, a comparison with those regularization techniques was not investigated.

We compared our ensembles and corresponding distilled networks with previously proposed methods. Several previous methods also employed ensembles containing networks with different^7^^,^^53^^,^^54^ or identical^48^ architectures. Knowledge distillation could have also been applied on these ensembles or previously proposed methods could have been added to our ensemble or used as architecture for the distilled network. However, the methods that participated in the challenges were often developed and optimized for a specific dataset, and therefore, a good performance might be data-dependent. In this work, we aimed to develop a robust automatic segmentation method that performed well on diverse datasets. For all datasets, our ensembles and corresponding distilled networks outperformed or obtained a performance close to previously proposed methods. This shows that our ensembles and subsequent knowledge distillation could be applied for automatic segmentation of structures of interest in medical images, differing in image modality, image dimensionality, and anatomical coverage.

## 6 Conclusion

The application of knowledge distillation allows for obtaining a single, smaller, and faster network for the segmentation of structures of interest in medical images. Employing an ensemble of networks with diverse architectures and loss functions does not necessary result in better performance compared with a more uniform ensemble. However, both types of ensembles outperform individual networks. Subsequent knowledge distillation of an ensemble results in a network that outperforms separate networks and furthermore could make the application of ensembles suitable in clinical practise.

## Source code

The code for training segmentation models, creating ensembles, and performing knowledge distillation is available at https://github.com/qurAI-amsterdam/ensemble-knowledge-distillation.
